# Clinician-assessed NYHA and EHRA symptom classifications only moderately reflect patient-reported quality of life in heart failure and atrial fibrillation

**DOI:** 10.1007/s12471-026-02051-9

**Published:** 2026-07-06

**Authors:** Jeroen A. A. van de Pol, Frederique J. Hafkamp, Pepijn H. van der Voort, Johannes C. Post, Sylvie F. A. M. S. de Jong, Monica Monroy, Sabine C. M. Eijsbouts, Ruud F. Spee, Arjen R. T. van de Ven, Boudewijn Klop

**Affiliations:** 1 Department of Electrical Engineering (SPS group), Eindhoven University of Technology (TU/e), Eindhoven, The Netherlands; 2 Netherlands Heart Network, Eindhoven, The Netherlands; 3 Department of Cardiology, Catharina Hospital, Eindhoven, The Netherlands; 4 Department of Cardiology, Elkerliek Hospital, Helmond, The Netherlands; 5 Department of Cardiology, Máxima Medical Center, Veldhoven, The Netherlands; 6 Department of Cardiology, Anna Hospital, Geldrop, The Netherlands

**Keywords:** Atrial Fibrillation, Heart Failure, Quality of Life, Physician-Patient Relations, Severity of Illness Index, Patient Outcome Assessment

## Abstract

**Background:**

Clinician-assessed symptoms are expected to agree with patient-perceived symptoms. However, patients may value the impact of symptoms on their health-related quality of life (HRQoL) differently from clinicians. This study aimed to evaluate the relationship between patient-perceived HRQoL and clinician-assessed symptom severity in patients with Heart Failure (HF) and Atrial Fibrillation (AF).

**Methods:**

Newly diagnosed HF and AF patients were prospectively included. Information on symptoms and HRQoL was collected at diagnosis and after 12 months during routine HF- and AF-outpatient clinic visits. Symptom severity was assessed by clinicians using the NYHA classification for HF and EHRA score for AF. HRQoL was assessed using the CaReQol-CHF (HF) and AFEQT (AF).

**Results:**

Complete baseline and 12-month follow-up information was available for 254 HF patients and 765 AF patients. Moderate correlations were observed between NYHA classification and CaReQoL-CHF at diagnosis and follow-up (Spearman’s ρ: HF_social_: 0.202 and 0.426, HF_physical_: 0.317 and 0.421, respectively), and between EHRA score and AFEQT (Spearman’s ρ: −0.363 and −0.305). Clinician-assessed improvement in HF or AF symptoms from diagnosis to 12 months was associated with patient-reported improvement in HRQoL (HF_social_: F(250.2) = 3.11, *p* < 0.046; HF_physical_: F(251.2) = 11.19, *p* < 0.001; and AF: F(762.2) = 25.86, *p* < 0.001). When clinicians reported unchanged or worsening symptoms, no significant HRQoL changes were reported by patients.

**Conclusion:**

Clinician-assessed symptoms correlate moderately with patient-reported HRQoL, with large inter-individual variation in HRQoL. NYHA and EHRA classifications may not accurately reflect patient-perceived burden of disease over time. Incorporating patient-reported outcomes into routine care may better guide treatment, improve the identification of patients’ needs, and support informed clinical and shared decision-making.

**Supplementary Information:**

The online version of this article (10.1007/s12471-026-02051-9) contains supplementary material, which is available to authorized users.

## What’s new?

- Clinician-assessed symptoms correlate moderately with patient-reported health-related quality of life in this allcomers nurse-led outpatient clinic in the Netherlands.
- Change in clinician-assessed symptoms only agrees with the change in patient-reported health-related quality of life in less than half of heart failure and atrial fibrillation patients in this nurse-led outpatient clinic in the Netherlands.
- There are no clear demographic or clinical characteristics across heart failure and atrial fibrillation that explain the difference in perceived trajectory (improvement and/or worsening of symptoms and health-related quality of life) between clinicians and patients in this allcomers nurse-led outpatient clinic in the Netherlands.

## Introduction

Heart failure (HF) and atrial fibrillation (AF) are among the most prevalent cardiac conditions, with prevalence expected to rise due to an aging population [1, 2]. Both are associated with functional impairment and reduced health-related quality of life (HRQoL) compared to the general population, driven by physical symptoms (e.g., dyspnea, fatigue) and psychosocial distress (e.g., anxiety, depression) [3, 4]. This impacts not only patients, but also healthcare systems, as lower HRQoL correlates with increased emergency visits, hospitalizations, and healthcare utilization [3, 5, 6].

Although survival in HF and AF has remained stable due to advances in care, improving daily functioning and symptom relief has become a central treatment goal [7]. Effective symptom management begins with assessing the extent to which symptoms limit daily life [8]. In practice, physicians use the New York Heart Association (NYHA) class for HF and the European Heart Rhythm Association (EHRA) score for AF, as recommended by clinical guidelines [9, 10].

However, these clinician-assessed tools may lack objectivity. Studies report poor inter-rater agreement and frequent discrepancies between physician and patient assessments of health status, with both over- and underestimation of impairment [11–16]. This inconsistency partly stems from the subjective nature of symptoms, which reflect not only physical limitations but also their broader impact on daily life [15]. Such discrepancies may lead to undertreatment, especially when patients are deemed asymptomatic, resulting in reduced therapy and poorer outcomes [17]. The way patients experience their health status is inherently subjective and may differ depending on illness perception, difficulties in self-assessment, and emotional adaptation to the diagnosis. Both clinician-assessed classifications and patient-reported outcome measures (PROMs) aim to evaluate the impact of symptom burden. However, both capture different, but complementary, constructs and perspectives.

This underscores the importance of standardized PROMs to assess symptom burden as experienced by patients. Disease-specific tools like the Care Related Quality of Life for Chronic Heart Failure Questionnaire (CaReQoL-CHF) [18] and the Atrial Fibrillation Effect on QualiTy-of-Life (AFEQT) questionnaire [19] capture HRQoL from the patient’s perspective and are validated for HF and AF, respectively [17, 20].

Yet, PROMs are underused in clinical practice due to time constraints, and evidence linking self-reported HRQoL with clinician-rated NYHA/EHRA scores remains limited [16]. This prospective observational study, aimed to evaluate how patients perceive the impact of HF and AF on HRQoL at diagnosis and after 12 months, and how these perceptions align with clinician-reported functional status in routine care.

## Abbreviated methods section

This prospective observational study was conducted within the Netherlands Heart Network (NHN), a regional collaboration of primary, secondary, and tertiary care providers in the Southeast of the Netherlands. The NHN aims to improve patient outcomes by standardizing and optimizing care pathways for common cardiovascular conditions. In this context, regional care standards for heart failure (HF) and atrial fibrillation (AF) have been implemented across four participating hospitals [21, 22].

### Participants

Patients were consecutively enrolled at the outpatient HF and AF clinics between June 2015 and February 2023 for HF, and between November 2014 and February 2023 for AF. Inclusion criteria were age ≥ 18 years and a recent or new diagnosis of non-valvular HF or AF. Patients had to be able to understand Dutch and provide informed consent. At baseline (T0), data were collected during routine nurse-led consultations, which included patient education and structured registration of demographic, clinical, and lifestyle characteristics in the electronic medical record. Follow-up data were collected at 12 months (T1) through outpatient visits or telephone consultations.

### Patient-reported health-related quality of life

Patient-reported HRQoL was assessed using validated disease-specific instruments: the Care-Related Quality of Life for Chronic Heart Failure Questionnaire (CaReQoL-CHF) for HF [18] and the Atrial Fibrillation Effect on QualiTy-of-Life (AFEQT) questionnaire for AF [19]. Questionnaires were completed at baseline and sent again at 12 months. From these, domain scores and overall HRQoL scores were derived. In the current study, only selected domains were included in analyses: physical and social-emotional limitations for HF, and the overall AFEQT score (excluding treatment satisfaction) for AF.

### Clinician-assessed functional impairment

Functional impairment was assessed by clinicians using guideline-recommended classification systems: the New York Heart Association (NYHA) classification for HF and the European Heart Rhythm Association (EHRA) score for AF. The NYHA classification system is routinely used to assess the severity of functional limitation of HF patients (NYHA I ‘no limitations of physical activity’; NYHA II ‘slight limitation of physical activity’; NYHA III ‘severe limitation of physical activity’; NYHA IV ‘inability of physical activity’). AF-related clinician-reported functional impairment was assessed by the (unmodified) EHRA symptom classification. The classification assesses the presence of symptoms and their effect on daily activities (EHRA I ‘no symptoms’; EHRA II ‘mild symptoms’; EHRA III ‘severe symptoms’; and EHRA IV ‘disabling symptoms’). The unmodified EHRA score was used since the study was designed before the introduction of the modified EHRA score in 2014.

Change in HRQoL and functional status was calculated over 12 months and categorized into trajectories (improvement, no change, deterioration) using an anchor-based method for the CaReQoL-CHF, and a minimal clinically important difference (MCID) of 5 for AFEQT, similar to the method of Holmes et al. [23] Agreement between HRQoL and functional impairment trajectories was assessed, and discrepancies were classified as overestimation or underestimation of impairment by clinicians. Multivariable regression analyses examined associations between patient characteristics and disagreement in trajectories. Full methodological details, including outcome definitions and statistical procedures, are provided in the Electronic Supplementary Materials [ESM].

## Results

### Sociodemographic characteristics

A total of 652 HF patients had complete information on HRQoL and NYHA at baseline, of whom 254 had complete 12-month follow-up data for either physical or social-emotional HRQoL and NYHA classification. The mean age of patients with available baseline NYHA and CaReQoL scores was 75 (± 10.2) years, and 59.4 % were male. 1942 AF patients had complete information on HRQoL and EHRA at baseline, 798 AF patients had complete information on HRQoL at 12 months, and 765 had complete data for both baseline and 12-month follow-up. Mean age was 69.7 (± 9.7) years, with 59.0 % male.

### Baseline clinical characteristics

Most HF patients (88 %) were classified as NYHA class II or III, indicating at least slight or severe limitations in daily living (Tab. 1). In contrast, most AF patients (84 %) were classified as EHRA 1 or 2, reflecting no or mild symptoms (Tab. 1). Among HF patients, 53 % had HFrEF (LVEF ≤ 40 %), and 48.5 % had concomitant AF. In AF patients, 60 % had paroxysmal AF, and 4 % had coexisting HF. Clinical characteristics differed significantly across NYHA and EHRA categories, including age, sex, and comorbidities (Tab. 1).

**Table 1:** Baseline characteristics of included heart failure patients (*N* = 652) and atrial fibrillation patients (*N* = 1942).

	***Heart Failure***				
*N,* (%)	*NYHA I* *N* = 61 (9.4 %)	*NYHA II* *N* = 317 (48.6 %)	*NYHA III* *N* = 258 (39.6 %)	*NYHA IV* *N* = 16 (2.5 %)	*p‑value*
Age, mean (SD)	74.5 (10.2)	74.2 (10.3)	76.7 (9.8)	72.3 (12.9)	0.017
Male	46 (75.4)	190 (59.9)	139 (53.9)	12 (75.0)	0.010
Type of HF					0.938
– HFrEF (≤ 40 % LVEF)	35 (57.4)	170 (55.0)	130 (55.1)	9 (60.0)	
– HFmrEF (40–49 % LVEF)	13 (21.3)	87 (28.4)	63 (26.7)	4 (26.7)	
– HFpEF (≥ 50 % LVEF)	13 (21.3)	52 (17.0)	43 (18.2)	2 (13.3)	
BMI, mean (SD)	26.0 (3.3)	26.7 (4.8)	27.7 (6.6)	25.8 (5.3)	0.145
Diabetes Mellitus	7 (14.0)	72 (26.8)	69 (28.7)	1 (9.1)	0.095
OSAS	2 (3.3)	28 (8.9)	28 (11.0)	1 (6.3)	0.301
Hypertension	27 (44.3)	153 (48.3)	156 (60.7)	7 (43.8)	0.010
Atrial fibrillation	35 (57.4)	136 (43.2)	140 (54.5)	4 (25.0)	<0.005
Malignancy	11 (18.0)	67 (21.2)	65 (25.3)	1 (6.3)	0.205
Chronic pulmonary disease	6 (9.8)	56 (17.8)	40 (15.6)	3 (18.8)	0.459
Cerebrovascular accident	3 (4.9)	18 (5.7)	24 (9.4)	1 (6.3)	0.678
Thyroid disease	1 (1.8)	20 (6.5)	17 (6.7)	0 (0)	0.678
Left bundle branch block	20 (35.1)	65 (25.1)	66 (27.3)	5 (38.5)	0.208
	***Atrial fibrillation***				
*N,* (%)	*EHRA 1* *N* = 850 (43.8 %)	*EHRA 2* *N* = 775 (39.9 %)	*EHRA 3* *N* = 233 (12.0 %)	*EHRA 4* *N* = 84 (4.3 %)	*p‑value*
Age, mean (SD)	70.7 (9.2)	68.9 (10.1)	68.8 (10.3)	70.2 (9.6)	<0.001
Male	577 (67.9)	423 (54.6)	105 (45.1)	40 (47.6)	<0.001
BMI, mean (SD)	28.0 (5.1)	27.6 (5.0)	27.7 (5.1)	26.5 (5.0)	0.077
CHADS_VASC ≥ 2	644 (76.1)	546 (70.5)	177 (76.2)	65 (77.4)	0.048
HAS_BLED ≥ 2	383 (48.7)	360 (47.7)	102 (44.7)	34 (42.5)	0.574
Diabetes Mellitus	132 (15.5)	78 (10.0)	25 (10.8)	10 (11.9)	0.008
OSAS	66 (43.7)	60 (40.0)	21 (12.1)	4 (4.3)	0.678
Hypertension	466 (54.9)	421 (54.3)	136 (58.6)	44 (52.4)	0.657
Type of AF					<0.001
– Paroxysmal	424 (49.9)	530 (68.4)	162 (69.5)	58 (69.0)	
– Persistent	317 (37.3)	199 (25.7)	58 (24.9)	22 (26.2)	
Heart Failure (≤ 40 % LVEF)	33 (3.9)	36 (4.6)	8 (3.4)	7 (8.3)	<0.001
Malignancy	112 (13.2)	84 (10.9)	29 (12.4)	13 (15.5)	0.405
Chronic lung disease	78 (9.2)	93 (12.0)	19 (8.2)	21 (25.0)	<0.001
Location of diagnosis					0.885
– General Practitioner	235 (27.6)	215 (27.7)	59 (25.6)	23 (27.4)	
– Hospital	604 (71.1)	547 (70.6)	171 (73.4)	61 (72.6)	

### Patient-reported health-related quality of life

At baseline, HF patients reported a median CaReQoL score of 3.50 (2.80–4.17) for physical impairment and 2.30 (1.70–3.00) for social-emotional impairment (scale 1–5, higher meaning worse HRQoL) (ESM Tab S1). After 12 months, both domains showed improvement: 3.00 (2.20–3.80) and 1.70 (1.20–2.50), respectively. The minimal clinically important difference (CID) was −0.458, which reflected the mean change in CaReQoL-CHF for all patients with a 1 NYHA class change (improvement or deterioration).

AF patients had a baseline median overall AFEQT score of 75.9 (57.4–89.8) (scale 0–100, higher meaning better HRQoL; ESM Tab S2). Domain-specific baseline scores were 83.3 (62.5–95.8) for symptoms, 72.9 (45.8–93.8) for daily activity, and 80.6 (63.9–94.4) for treatment concerns. At 12 months, all domains improved: overall score increased to 85.2 (69.6–95.4), with domain scores of 91.7 (78.5–100.0), 81.3 (58.3-97.9), and 91.7 (58.3–97.9), for symptoms, daily activity, and treatment concerns, respectively.

### Correlation between HRQoL and clinician-assessed symptom scores

**Fig. 1:**
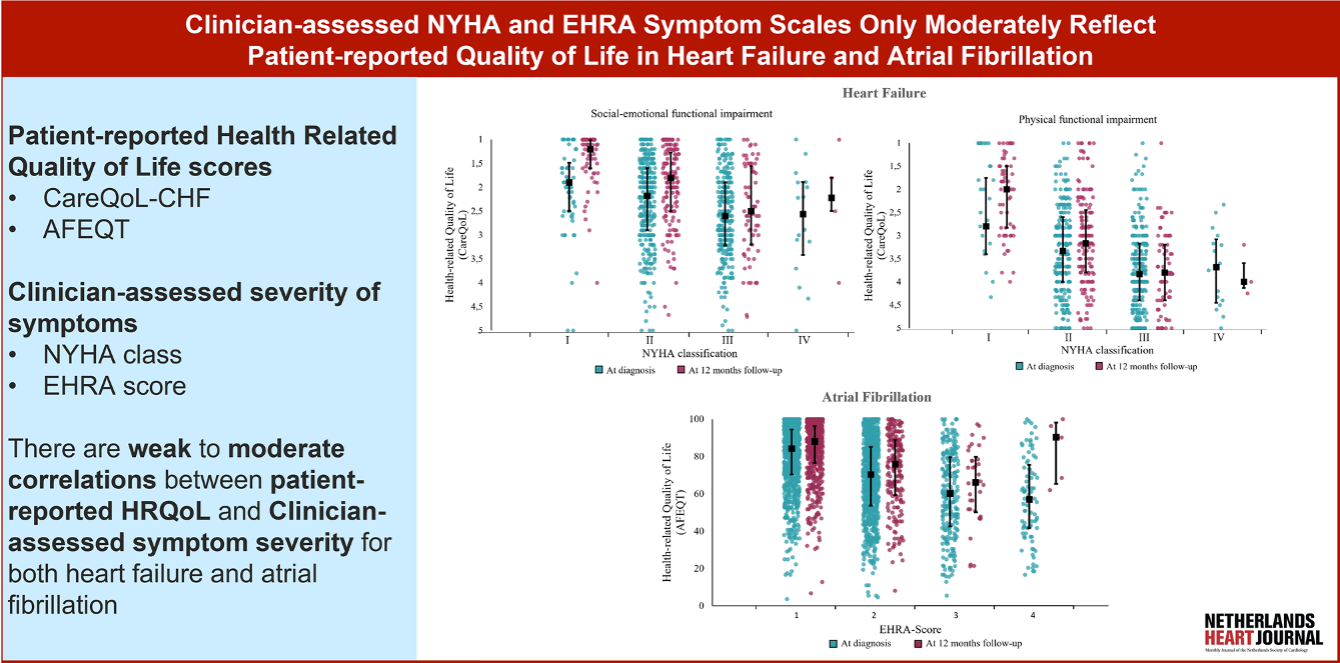
Infographic showing the correlation between patient-reported health related quality of life and clinician-assessed severity of symptoms for heart failure and atrial fibrillation

**Fig. 2:**
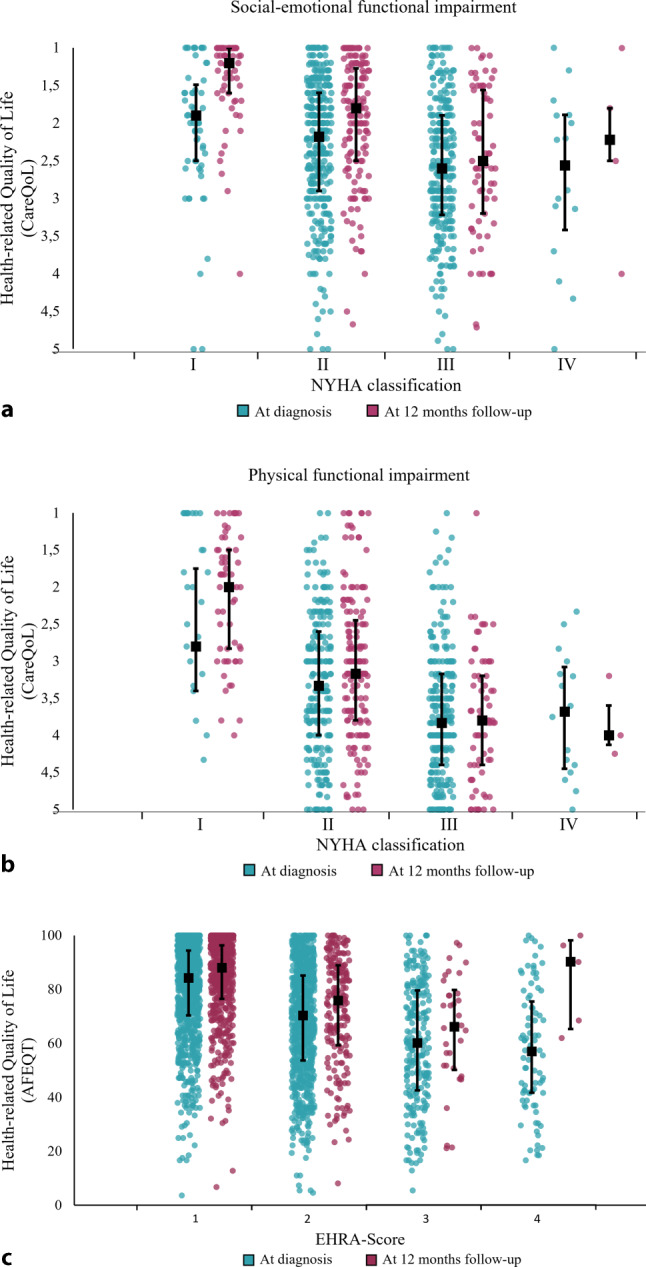
**a**–**c** Scatter plot between quality of life and functional impairment for heart failure (**a**, **b**) and atrial fibrillation (**c**) at diagnosis and 12 months of follow-up. Small dots depict individual patients’ answers. Squares depict group means

In HF patients, social-emotional HRQoL showed weak but significant correlations with NYHA classification (baseline: spearman’s *p* = 0.202, *p* < 0.001; follow-up: *p* = 0.426, *p* < 0.001; Fig. 1, 2a). Physical HRQoL showed a moderate correlation with NYHA class at baseline (*p* = 0.317, *p* < 0.001) and follow-up (*p* = 0.421, *p* < 0.001; Fig. 2b). HRQoL decreased with increasing NYHA class, and after 12 months, all NYHA groups except NYHA IV showed improvement (ESM Tab S1).

In AF patients, moderate correlations were found between AFEQT scores and EHRA class at both baseline (*p* = −0.363, *p* < 0.001) and follow-up (*p* = −0.305, *p* < 0.001; Fig. 2c). The strongest correlation was seen in the symptom domain (baseline: *p* = −0.378; follow-up: *p* = −0.350), while the weakest was for daily activity (baseline: *p* = −0.287; follow-up *p* = −0.230). HRQoL decreased with higher EHRA class. HRQoL decreased with increasing EHRA class, and all EHRA groups showed improved QoL over 12 months (ESM Tab S2).

### Agreement between patient-reported HRQoL and symptom trajectory

One-way ANOVA revealed significant differences in patient-reported HRQoL change across clinician-assessed symptom trajectory groups (i.e., improvement, no change or deterioration) in both HF and AF patients (HF: physical F (251.2) = 11.19, *p* < 0.001; social-emotional F (250.2) = 3.11, *p* = 0.046; AF: F (762.2) = 25.86, *p* < 0.001) (Tab. 2).

**Table 2:** Median change and interquartile range in overall quality of life by change in trajectory for severity of functional impairment (NYHA classification/EHRA score).

	Change in CareQoL-CHF					Change in AFEQT
Impairment trajectory (NYHA/EHRA)	*N*, %	Physical functional impairment Median (Q1–Q3)	*N*, %	Social-emotional functional impairment Median (Q1–Q3)	*N*, %	Median (Q1–Q3)
Improvement	99 (39.0 %)	−0.33 (−1.00; 0.30)	101 (39.9 %)	−0.32 (−1.10; 0.00)	277 (36.2 %)	12.7 (0.0; 27.8)
No change	127 (50.0 %)	−0.07 (−0.53; 0.47)	123 (48.6 %)	−0.15 (−0.85; 0.39)	444 (58.0 %)	2.8 (−3.7; 11.1)
Deterioration	28 (11.0 %)	−0.17 (−0.92; 0.52)	29 (11.5 %)	−0.20 (−0.90; 0.10)	44 (5.8 %)	−0.5 (−11.3; 12.4)

In HF, all groups reported HRQoL improvement, but the largest improvement was seen in patients whose NYHA class also improved. Post-hoc analysis showed this group differed significantly from those with unchanged NYHA class (physical: 95%CI −0.86; −0.26, *p* < 0.001; social: 95%CI −0.59;−0.00, *p* = 0.049), but not from those with NYHA deterioration.

In AF, HRQoL improvements aligned more closely with EHRA change. Patients with improved EHRA scores had significantly greater HRQoL improvement than those with no change (95 % CI 6.18–13.38, *p* < 0.001) or deterioration (95 % CI 6.38–21.63, *p* < 0.001).

**Fig. 3:**
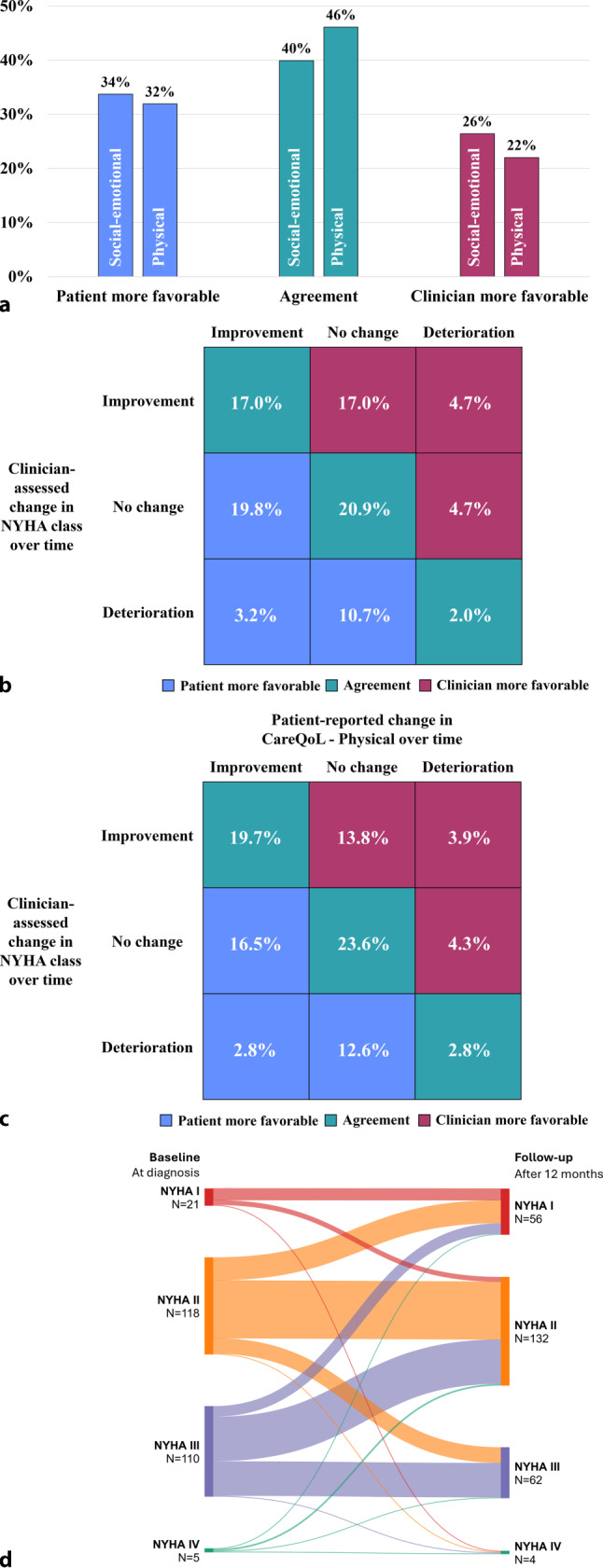
**a** Agreement between patient-reported change in QoL compared to the clinician-assessed change in functional impairment over 12 months for HF. **b**, **c** Detailed insights in reported trajectories for social-emotional QoL for HF (**a**) and the physical QoL for HF (**b**). **d** Detailed insights in reported trajectories for change in NYHA class for HF

**Fig. 4:**
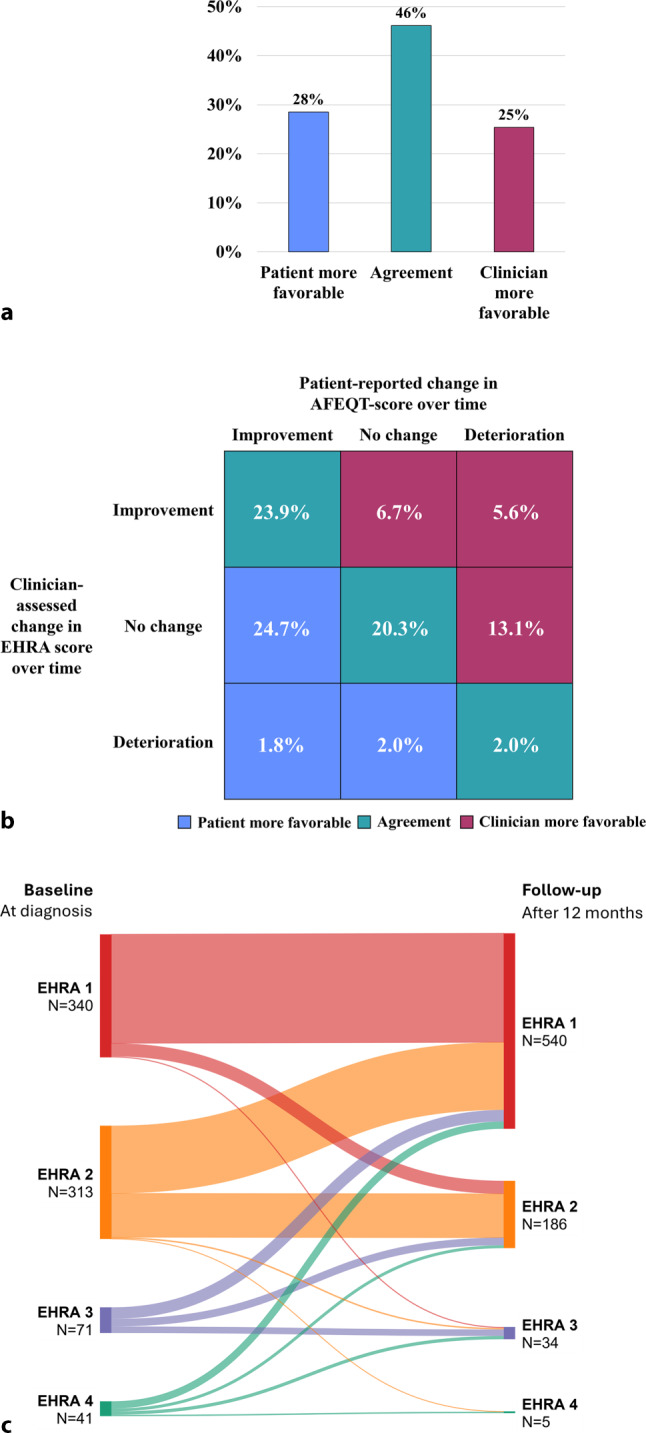
**a** Agreement between patient-reported change in QoL compared to the clinician-assessed change in functional impairment over 12 months for AF. **b** provides detailed insights in reported trajectories for QoL for AF. **c** provides detailed insights in reported trajectories for change in EHRA score for AF

Agreement between patient-reported HRQoL and clinician-assessed symptom trajectory was observed in 34–46 % of HF cases and 46 % of AF cases (Fig. 3a and 4a). In 32–40 % of HF and 28 % of AF cases, patients reported HRQoL improvement without clinician-assessed symptom change. Conversely, in 22–26 % (HF) and 28 % (AF), clinicians reported improvement not reflected in patient-reported HRQoL. More detailed trajectories are presented in Fig. 3b, c and 4b. EHRA and NYHA trajectories are shown in detail in Fig. 3d and 4c.

### Factors associated with over- and underestimation of trajectories

Multivariable regression identified several predictors that may have contributed to overestimation (i.e., the clinician-assessed symptom trajectory indicated a more favorable outcome, compared to the HRQoL trajectory) or to underestimation (i.e., the clinician-assessed symptom trajectory indicated a less favorable trajectory, compared to the HRQoL trajectory). Among HF patients, clinicians were less likely to overestimate the symptom trajectory, compared to the HRQoL trajectory if the patient had concomitant AF (OR 0.52, 95 % CI 0.28–0.95, *p* = 0.034), while clinicians were more likely to underestimate the symptom trajectory in patients with a history of CVA (OR 3.22, 95%CI 1.01–10.27, *p* = 0.048). No significant predictors were found for social-emotional HRQoL over- and underestimation.

In AF patients, older age (per 5–year increment) was associated with higher odds of clinicians overestimating the symptom trajectory compared to the patient-reported HRQoL trajectory (OR 1.11, 95 % CI 1.006–1.24, *p* = 0.038). In addition, prior or present malignancy (OR 1.74, 95 % CI 1.04–2.90, *p* = 0.034) was associated with overestimation. No predictors remained significant for underestimation.

## Discussion

This study found moderate correlations between patient-reported HRQoL and clinician-assessed functional impairment in HF and AF patients. At both diagnosis and 12-month follow-up, wide variability in HRQoL was observed even among those classified as asymptomatic. Less than half of patients showed alignment between changes in HRQoL and changes in NYHA or EHRA scores. Notably, comorbid AF and history of CVA in HF were associated with over- and underestimation, respectively, of disease trajectories by clinicians. Age and malignancy in AF were associated with overestimation of trajectories. Overall, this study highlights that the clinician-assessed classifications on functional impairment in HF and AF do not accurately reflect changes in QoL after treatment as reported by the patient.

Despite growing emphasis in guidelines on incorporating patient-reported outcomes in HF and AF care, clinical use remains limited [24, 25]. Clinicians typically use NYHA and EHRA scores to describe the functional status of HF and AF patients. However, these assessments may be influenced by clinical impressions, time constraints, or cognitive biases, and often fail to capture subjective experiences like fatigue or anxiety [8, 15]. Prior research shows that NYHA class correlates poorly with objective performance measures like the 6‑minute walk test, and physician agreement on NYHA classification is limited (56 %) [26, 27]. Inter-rater variability is especially high in milder symptom groups (NYHA I–II, EHRA 1–2) [28, 29]. However, it should be clarified that the clinician-assessed functional impairment using the NYHA and EHRA classification does not aim to capture emotional, social, or psychological components of HRQoL. Therefore, these constructs partially overlap with patient-reported HRQoL and moderate correlation coefficients between them are not unexpected.

Our findings confirm this issue, showing substantial variation in patient-reported HRQoL even among patients rated as asymptomatic by their clinician. Agreement on change over time was also low, supporting prior studies showing that meaningful changes in HRQoL are not always reflected in clinician ratings [8, 30]. Greene *et al.* found that HRQoL improved in many HF patients without corresponding NYHA change, suggesting limited sensitivity of NYHA to treatment response [30]. Potential causes of discordance may include demographic or psychosocial factors. While some studies have found associations with sex, income, or race/ethnicity, we found a few significant predictors, namely AF and CVA in HF, and age and malignancy in AF [30]. This suggests that discordance may be more related to communication, perception, or measurement differences rather than patient characteristics alone.

Some argue that functional status should be assessed directly by patients, as HRQoL encompasses not only physical limitations but also emotional and social aspects [7, 31]. HRQoL is a strong predictor of hospitalization, costs, and mortality [32–34]. It also more reliably detects clinically meaningful changes than clinician-assessed health status [30]. Yet, relying solely on patient reports may also have limitations. Patients with multimorbidity may struggle to differentiate symptoms belonging to HF or AF, or other comorbidities, and daily fluctuations in symptoms can be challenging for patients to detect [35].

A combined approach may be most beneficial. Integrating HRQoL tools like CaReQoL and AFEQT, alongside NYHA and EHRA scores, could provide a more comprehensive view of the patient’s health. This may be important both at the start of treatment and during the subsequent treatment process. Prior research shows that when clinicians have access to HRQoL data, discordance with patient perception decreases [16, 36]. This approach can enhance clinical and shared decision-making and support patient-centered care.

Several limitations should be acknowledged. Most patients in this study had mild to moderate symptoms, limiting generalizability to more severe cases. The unmodified EHRA score was used, which lacks the differentiation present in the modified version between mild but troublesome and non-troublesome symptoms. The unmodified EHRA might have limited our ability to accurately distinguish patients in these groups, which are known to be hard to differentiate. Additionally, we lacked data on clinician sex, which may influence the clinicians’ assessment, especially given evidence suggesting that patient-clinician sex concordance affects patient experience and outcomes [37]. In addition, half of the participating hospitals had two nurses running the nurse-led clinic instead of one single nurse. This may have led to inter-observer variability as a confounder in the assessment of baseline and one-year follow-up NYHA class and EHRA scores in approximately a quarter of the patients. Unfortunately, we did not have data regarding the inter-observer variability between the nurses. Complete follow-up of HF- and AF-patients was limited, with more than 50 % of the patients lost to follow-up after one year. This was primarily attributable to a lack of response by patients to fill out and return the HRQol questionnaires at follow-up. This may have led to selection and attrition bias. In this study, we did not perform 6‑minute walk tests, which may have helped objectively assess the physical capacity of the HF and AF patients. This was because our observational study was conducted during routine clinical practice and the one-year follow-up consisted of telephone consultations, with patients returning their HRQoL questionnaires by mail. Lastly, for HF patients, the CareQol-CHF was used. This questionnaire has been developed based on existing international heart failure instruments and validated in the Netherlands [18]. This may limit direct extrapolation of our results in non-Dutch populations.

This study demonstrates moderate concordance between patient-reported HRQoL and clinician-assessed symptom severity in HF and AF. Less than half of the trajectories aligned over time, with notable variability in HRQoL even among patients rated as asymptomatic. These findings highlight the limitations and partial agreement of NYHA and EHRA scores in capturing the full patient experience. The complementary incorporation of validated patient-reported outcomes into routine care may better guide treatment, more accurately identify patients’ needs, and support better-informed clinical and shared decision-making.

## Supplementary Information

Supplementary methods

Table S1—Median with interquartile range for CaReQoL-score for each NYHA-impairment severity category in HF patients.

Table S2—Median with interquartile range for AFEQT-score for each EHRA-impairment severity category in AF patients.

## Data Availability

The datasets generated during and/or analysed during the current study are available from the corresponding author upon reasonable request.
