# Supplementary material for: Clinician-assessed NYHA and EHRA symptom classifications only moderately reflect patient-reported quality of life in heart failure and atrial fibrillation
Source: Neth Heart J. 2026 Jul 6;34(7-8):274–82. doi: 10.1007/s12471-026-02051-9 (PMC13375993; doi:10.1007/s12471-026-02051-9)
Supplement: Supplementary file 1 — Supplementary methods [file 12471_2026_2051_MOESM1_ESM.docx]

**Clinician-assessed NYHA and EHRA Symptom Classifications Only Moderately Reflect Patient-reported Quality of Life in Heart Failure and Atrial Fibrillation**

J.A.A. van de Pol^*^, F.J. Hafkamp^*^, P.H. van der Voort, J.C. Post, S.F.A.M.S. de Jong, M. Monroy, S.C.M. Eijsbouts, R.F. Spee, A.R.T. van de Ven, B. Klop

**Supplementary methods:**

This study was performed within the context of the Netherlands Heart Network (NHN). The NHN is a joint-effort of healthcare providers in primary, secondary and tertiary care collaborating and aiming to continuously improve patient value for patients in the Southeast of the Netherlands by optimizing the complete healthcare chain. To this end, regional care standards have been implemented to standardize and optimize care for HF- and AF-patients visiting the outpatient clinics in the hospitals embedded within the NHN. Details on the methods of the NHN can be found elsewhere.^1, 2^

*Participants*

Patients were derived from a cohort of patients newly or recently diagnosed with AF or HF within one of the four hospitals embedded within the NHN between June 2015 and February 2023 for HF and November, 2014 and February, 2023 for AF. Patients were consecutively included at the outpatient AF and HF clinics of the four hospitals when they were ≥18 years, newly or recently diagnosed with (non-valvular) AF or HF, and provided informed consent. Patients were excluded from the study if they did not speak Dutch or if they were unable to understand or complete the informed consent.

*Procedure*

During 40-60 minute patient consultation sessions trained AF- and HF-nurses provided patient education and registered data on baseline sociodemographic variables, clinical variables, comorbidities and prior events, and lifestyle of patients in the patients’ electronic medical records (EMR). Baseline data were collected at time of diagnosis during the visit to the outpatient clinic (T0). Outcome data (e.g., hospitalization and mortality) were collected at 12 months after diagnosis either through a visit to the outpatient clinic or through telephonic consultation (T1).

*Patient-reported quality of life*

Patients completed the CaReQoL-CHF^3^ (HF) or AFEQT^4^ (AF) questionnaire at baseline to assess their perceived HRQoL. HRQoL questionnaires were completed either during the visit to the HF- or AF outpatient clinic, or were completed and returned by mail after the visit. HRQoL questionnaires, including an answer envelope, were sent to the patient by mail after 10 to 12 months of follow-up. In this study, the CaReQoL for chronic heart failure (CaReQoL-CHF) was employed, which features 20 items across three domains, namely social-emotional limitations, physical limitations and being in safe hands. For all patients that completed at least half of the items within a domain an average sub-score was calculated. The CaReQoL-CHF uses a 5-point Likert response scale per item with 1 denoting a low impact on HRQoL and 5 denoting a high impact on HRQoL of a particular item. The AFEQT questionnaire was used for AF patients. The AFEQT questionnaire is a validated and reliable questionnaire featuring 20-items across 4 subdomains, including symptoms, daily activities, treatment concerns and treatment satisfaction on a 7-point Likert response scale. The overall AFEQT score is calculated based on the answers from the first three subdomains (18 questions) and ranges from 0 (severe impairment/low HRQoL) to 100 (no impairment/high HRQoL).

The subscales ‘being in safe hands’ for HF and ‘treatment satisfaction’ for AF were not considered in this study. For HF, the subscales physical and social-emotional limitations were used in analyses. For AF, an overall AFEQT score was used.

*Clinician-assessed functional impairment*

The clinician-assessed functional impairment was reported through the use of the NYHA classification in HF-patients. The NYHA classification system is routinely used to assess the severity of functional limitation of HF patients (NYHA I ‘no limitations of physical activity’; NYHA II ‘slight limitation of physical activity’; NYHA III ‘severe limitation of physical activity’; NYHA IV ‘inability of physical activity’)^5, 6^. AF-related clinician-reported functional impairment was assessed by the (unmodified) EHRA symptom classification. The classification assesses the presence of symptoms and their effect on daily activities (EHRA I ‘no symptoms’; EHRA II ‘mild symptoms’; EHRA III ‘severe symptoms’ and EHRA IV ‘disabling symptoms’).

*Statistical analyses*

Baseline patient characteristics are presented as frequencies and percentages for categorical variables and means and SD or median and interquartile range for continuous variables. In addition, means and standard deviations and medians and interquartile ranges were employed in analyses on the patient-reported HRQoL and clinician-assessed severity of functional impairment. For all analyses on correlations Spearman’s ρ including the corresponding p-value was reported.

Change in HRQoL over 12 months was quantified by subtracting the HRQoL at diagnosis from the QoL after 12 months of follow-up. A minimal clinical important difference (CID) of 5 was used for the AFEQT questionnaire based on Holmes *et al.*^7^. For the CaReQoL-CHF this information was not available. Therefore, an anchor based method was used, similar to Holmes *et al.*. In short, the mean change was used to identify CIDs in CaReQoL-CHF after 12 months of follow-up. The CID was determined by assessing the mean change in CareQoL-CHF for all patients with a 1 NYHA class change (improvement or deterioration). An increase or decrease in HRQoL greater that the CID was categorized as improvement or deterioration.

Change in severity of functional impairment was determined by subtracting the NYHA classification (HF) or EHRA score (AF) at diagnosis from the NYHA classification or EHRA score after 12 months of follow-up. For both NYHA classification and EHRA score an improvement or deterioration of 1 was seen as clinically important. Trajectories were categorized as follows; 1, improvement; 0, no change; -1; deterioration). One-way ANOVA’s were employed to assess differences in QoL change across symptom trajectories. Scheffe’s method was used to perform post-hoc tests for multiple comparisons.

Agreement between change in functional impairment and HRQoL over 12 months was assessed using the aforementioned classification of disease trajectories. Trajectories were considered to be in agreement when both functional impairment and HRQoL were categorized as showing improvement, no change or deterioration. In instances of disagreement, where trajectories diverged, further subclassification was applied. If the clinician-reported symptom trajectory indicated a more favorable outcome, compared to the HRQoL trajectory (e.g. no change in HRQoL or HRQoL deterioration and NYHA/EHRA improvement), it was categorized as overestimation. If the symptom trajectory indicated a worse outcome than the reported HRQoL trajectory, it was categorized as underestimation.

Multivariable-adjusted linear regression models were employed to identify associations between patient characteristics and comorbidities and over- and underestimation of disease trajectories. Age (in 5 year increments) and sex (female/male) were included in all models, while covariates were tested using backwards elimination with a p-value threshold of 0.05. For HF, covariates considered were Body Mass Index (BMI; kg/m2), hypertension (yes/no), prior or present malignancy (yes/no), OSAS (yes/no), diabetes mellitus (yes/no), history of CVA (yes/no), thyroid disease (yes/no), COPD (yes/no), AF (yes/no), LVEF (yes/no) . For AF, covariates included BMI (kg/m2), hypertension (yes/no), prior or present malignancy (yes/no), heart failure (yes/no), OSAS (yes/no), diabetes mellitus (yes/no), history of CVA (yes/no), type of atrial fibrillation (paroxysmal/persistent), and treatment strategy (rate control/rhythm control).

Statistical analyses were performed using IBM SPSS Statistics version 28. A 0.05 level of significance was applied to evaluate statistical significance.

**References:**

1. Van Veghel H, Dekker L, Theunissen L, Janssen J, Burg M, Huijbers P, et al. Introducing a method for implementing value based health care principles in the full cycle of care: Using atrial fibrillation as a proof of concept. International Journal of Healthcare Management. 2022;15(1):1-9.

2. Theunissen L, Cremers H-P, Dekker L, Janssen H, Burg M, Huijbers E, et al. Implementing value-based health care principles in the full cycle of care: the pragmatic evolution of the Netherlands heart network. Circulation: Cardiovascular Quality and Outcomes. 2023;16(4):e009054.

3. Van Kessel P, de Boer D, Hendriks M, Plass AM. Measuring patient outcomes in chronic heart failure: psychometric properties of the Care-Related Quality of Life survey for Chronic Heart Failure (CaReQoL CHF). BMC health services research. 2017;17:1-7.

4. Spertus J, Dorian P, Bubien R, Lewis S, Godejohn D, Reynolds MR, et al. Development and validation of the Atrial Fibrillation Effect on QualiTy-of-Life (AFEQT) Questionnaire in patients with atrial fibrillation. Circulation: Arrhythmia and Electrophysiology. 2011;4(1):15-25.

5. Bennett JA, Riegel B, Bittner V, Nichols J. Validity and reliability of the NYHA classes for measuring research outcomes in patients with cardiac disease. Heart & Lung. 2002;31(4):262-70.

6. Zimerman A, da Silveira AD, Solomon SD, Rohde LE. NYHA classification for decision‐making in heart failure: Time to reassess? European Journal of Heart Failure. 2023;25(7):929-32.

7. Holmes DN, Piccini JP, Allen LA, Fonarow GC, Gersh BJ, Kowey PR, et al. Defining clinically important difference in the atrial fibrillation effect on quality-of-life score: Results from the outcomes registry for better informed treatment of atrial fibrillation. Circulation: Cardiovascular Quality and Outcomes. 2019;12(5):e005358.
