# Supplementary material for: Clinician-assessed NYHA and EHRA symptom classifications only moderately reflect patient-reported quality of life in heart failure and atrial fibrillation
Source: Neth Heart J. 2026 Jul 6;34(7-8):274–82. doi: 10.1007/s12471-026-02051-9 (PMC13375993; doi:10.1007/s12471-026-02051-9)
Supplement: Supplementary file 2 — Table S1—Median with interquartile range for CaReQoL-score for each NYHA-impairment severity category in HF patients. [file 12471_2026_2051_MOESM2_ESM.docx]

**Tab S1** Median with interquartile range for CareQoL-score for each NYHA-impairment severity category in HF patients.

|  |  |  | **Heart Failure** | | | |  |
| --- | --- | --- | --- | --- | --- | --- | --- |
|  |  | **NYHA** | **N, %** | **Physical**  Median (Q1-Q3) | **N, %** | **Social-emotional**  Median (Q1-Q3) |  |
| ***At diagnosis (T0)**** | | | | | | |  |
|  | | | | | | |  |
|  |  | I | 61 (9.4%) | 2.80 (1.75-3.40) | 59 (9.1%) | 1.90 (1.49-2.50) |  |
|  |  | II | 314 (48.3%) | 3.33 (2.60-4.00) | 314 (48.5%) | 2.18 (1.60-2.90) |  |
|  |  | III | 258 (39.8%) | 3.83 (3.17-4.40) | 258 (39.9%) | 2.60 (1.90-3.22) |  |
|  |  | IV | 16 (2.5%) | 3.68 (3.08-4.45) | 16 (2.5%) | 2.56 (1.89-3.42) |  |
| ***At 12 months follow-up (T12)**** | | | | | | |  |
|  | | | | | | |  |
|  |  | I | 60 (21.4%) | 2.00 (1.50-2.83) | 61 (21.6%) | 1.20 (1.00-1.60) | |
|  |  | II | 147 (52.3%) | 3.17 (2.45-3.80) | 147 (52.1%) | 1.80 (1.27-2.50) | |
|  |  | III | 70 (24.9%) | 3.80 (3.20-4.40) | 69 (24.5%) | 2.50 (1.56-3.20) | |
|  |  | IV | 4 (1.4%) | 4.00 (3.60-4.13) | 5 (1.8%) | 2.22 (1.80-2.50) | |

^*^*Calculations for individual subdomains were performed according to the CareQoL-CHF scoring guides.*
