# Supplementary material for: Clinician-assessed NYHA and EHRA symptom classifications only moderately reflect patient-reported quality of life in heart failure and atrial fibrillation
Source: Neth Heart J. 2026 Jul 6;34(7-8):274–82. doi: 10.1007/s12471-026-02051-9 (PMC13375993; doi:10.1007/s12471-026-02051-9)
Supplement: Supplementary file 3 — Table S2—Median with interquartile range for AFEQT-score for each EHRA-impairment severity category in AF patients. [file 12471_2026_2051_MOESM3_ESM.docx]

**Tab S2** Median with interquartile range for AFEQT-score for each EHRA-impairment severity category in AF patients.

|  |  |  | **Atrial fibrillation** | | | | |
| --- | --- | --- | --- | --- | --- | --- | --- |
| **EHRA** | | | **N, %** | **Overall**  Median (Q1-Q3) | **Symptoms**  Median (Q1-Q3) | **Daily activity**  Median (Q1-Q3) | **Treatment concern**  Median (Q1-Q3) |
| ***At diagnosis (T0)****^†^* | | | | | | | |
| 1  2  3  4 | | |  |  |  |  |  |
|  |  |  | 850 (43.8%) | 84.3 (70.4-94.4) | 91.7 (76.4-100.0) | 83.3 (58.3-97.9) | 88.9 (75.0-97.3) |
|  |  |  | 775 (39.9%) | 70.4 (53.7-85.2) | 75.0 (58.3-87.5) | 68.8 (41.7-89.6) | 77.8 (61.1-88.9) |
|  |  |  | 233 (12.0%) | 60.2 (42.6-79.6) | 62.5 (41.7-83.3) | 54.2 (27.1-79.2) | 70.8 (52.8-86.1) |
|  |  |  | 84 (4.3%) | 57.1 (41.7-75.5) | 70.8 (47.9-87.5) | 49.0 (21.9-67.7) | 68.1 (45.8-83.3) |
|  | | | | | | | |
| ***At 12 months follow-up (T12)****^†^* | | | | | | | |
|  |  |  |  |  |  |  |  |
| 1  2  3  4 | | | 564 (70.7%) | 88.0 (76.6-96.3) | 91.7 (83.3-100.0) | 83.3 (66.7-100.0) | 94.4 (83.3-100.0) |
|  |  |  | 195 (24.4%) | 75.9 (59.7-88.9) | 79.2 (58.3-91.7) | 72.9 (47.9-91.7) | 83.3 (63.9-97.2) |
|  |  |  | 34 (4.3%) | 66.2 (50.9-78.6) | 75.0 (58.3-87.5) | 54.2 (25.0-79.2) | 83.3 (61.1-88.9) |
|  |  |  | 5 (0.6%) | 90.2 (68.5-96.3) | 91.7 (83.3-100.0) | 81.0 (56.3-100.0) | 100.0 (83.3-100.0) |

*^*^Individual subscales may contain fewer responses due to insufficient information to score individual subdomains*

*^†^Calculations for individual subdomains and overall scores were performed according to the AFEQT scoring guides.*
